# Inclusion of Residual Tissue in Biobanks: Opt-In or Opt-Out?

**DOI:** 10.1371/journal.pbio.1001373

**Published:** 2012-08-07

**Authors:** Noor A. A. Giesbertz, Annelien L. Bredenoord, Johannes J. M. van Delden

**Affiliations:** Department of Medical Humanities, Julius Center, University Medical Center Utrecht, The Netherlands; University of Pennsylvania, United States of America

## Abstract

Residual samples are an important source of tissue for biobanks. They refer to leftover tissue that is obtained in the course of clinical care. Residual samples can be included through an opt-in method—that is, a person explicitly expresses consent to include residual tissue—or an opt-out method—that is, the tissue is stored unless a person explicitly refuses. At the moment there is a renewed interest in the appropriate method for the inclusion of residual samples in biobanks. The expansion of biobanks and rapid developments in biomedical research underscore the need to evaluate the proper procedure. In this article we revisit the arguments in favor and against opt-in and opt-out methods for residual tissue research. We conclude firstly that an opt-out method is only justifiable when certain conditions are met: (1) awareness has to be raised, (2) sufficient information has to be provided, and (3) a genuine possibility to object has to be offered. An opt-out procedure that fulfills these conditions can be called a “thick” opt-out method. As a consequence, the dichotomy between opt-in and opt-out is less stark than usually suggested, as both methods require a certain amount of effort. Secondly, we conclude that because of the diversity of tissue and research, not every situation can be treated alike. There are at least four situations that require opt-in procedures: (1) research with higher risks or increased burdens, (2) the use of controversial or high-impact techniques, (3) research on sensitive tissue types, and (4) research involving vulnerable patients. We suggest that further interdisciplinary debate should answer the question when to opt-in or when to opt-out.

## Abstract 2

This paper reviews the key arguments of the two predominant methods for the inclusion of human residual tissue in biobanks: opt-in and opt-out.

A biobank can be defined as a collection of human biological samples stored for medical-scientific research purposes, usually linked to phenotypic data [1],[2]. To collect material, biobanks have different strategies: they may collect tissue specifically for research purposes, but often contain *residual* samples as well. Residual samples refer to tissue that was taken in the course of clinical care and is leftover (e.g., a diagnostic biopsy or therapeutic removal of tissue). In many cases, the stored tissue will be most valuable for research when it remains linked to information about the person [3]–[5]. Therefore, the included samples will often be stored coded and consequently will not be anonymous—if complete anonymization would be possible at all [6]–[8].

Regarding the inclusion of residual tissue, two predominant methods can be discerned: *opting-in* and *opting-out*. In an opt-in scheme, a person explicitly expresses his or her consent. Contrarily, in an opt-out scheme inaction is treated as a signal of consent [9]. A strong consensus exists that opting-in is the preferred method to include people in clinical research [10]. The same counts when tissue is collected specifically for research, since research is the only reason for taking the sample. However, at the moment, there is no consensus about the most appropriate procedure to include residual samples. Both opt-in and opt-out methods are practiced and advocated [11]: some even argue that there is no consent required at all [12],[13].

The expansion of biobanks and the rapid developments in biomedical research (e.g., the emergence of whole genome sequencing (WGS) and the increase of personal information that can be derived from research [8]) emphasize the need to rethink the appropriate way to include residual tissue in biobanks [14],[15]. Therefore, we sought the key arguments in favor and against opt-in and opt-out methods for residual tissue research in the literature. The aim of the article is to present an overview of the arguments and to discuss them briefly. We focus on new residual tissue that is stored coded and will not review the subject of archival residual tissue—residual tissue that is already stored at this moment. We will conclude by offering some brief comments in order to take the current debate a step forward.

## Arguments in Favor of an Opt-Out Method for Inclusion of Residual Samples

### Scientific Advantage

Opt-out procedures for the inclusion of residual tissue in biobanks are associated with low refusal rates and therefore high participation rates [16],[17]. In order to generate scientifically valid results, sufficient numbers of samples are needed. People who are indifferent or do not mind participating in research, but are not willing to make an effort to explicitly consent, will be enrolled in an opt-out procedure. Conversely, they will not be included in an opt-in procedure.

However, it has been noticed that an opt-out method could, in theory, have a negative effect on participation rates as well. If people dislike the idea that their consent is not explicitly sought, it is possible they opt-out of participation because of public distrust or because their “gift” is taken for granted [9].

### Lower Financial Costs

An opt-out procedure is associated with lower costs [18],[19] compared to an opt-in procedure—which is generally costlier due to extensive procedures and lower participation rates [20],[21]. Money spent on the recruitment of participants cannot be used for other purposes. As a result the quality of the research may decrease or some projects may not be able to start at all.

However, whether an opt-out procedure indeed is considerably less costly depends on the way the formulated conditions are filled in. A very “thin” opt-out procedure, without adequate information provision or genuine possibilities to object, will certainly be less expensive. But when more demanding conditions are formulated, an opt-out procedure requires certain amounts of efforts and thus money as well [22].

### Moral Duty to Participate

The aim of conducting biomedical research is to generate biomedical knowledge, which is a benefit as such [2],[23],[24] and may eventually also prevent serious harm [25],[26]. Therefore, with an appeal to principles such as beneficence, solidarity, and reciprocity, participation in biomedical research can be considered a moral duty [25],[27]–[29]. Solidarity calls for mutual support [30] and reciprocity appeals to a duty to contribute to the development of biomedical knowledge since we all benefit from it [25],[31]. Although this duty would exist both with an opt-in and an opt-out procedure, it can nevertheless provide an argument for a less demanding procedure for the inclusion of residual tissue, like the opt-out procedure. After all, when there would be a moral duty to allow inclusion of residual tissue, it is reasonable to adopt a system where participation is the starting point or default position. Some even consider it an argument for not obtaining consent at all [12],[27].

However, several important objections have been made against this duty [32],[33]. For example, there are many actions that would benefit others or could rescue them (e.g., donate money for food). Priority or a special status for participation in biomedical research, compared to other goals, has not been shown. Another objection that has been made is that people already contribute to biomedical research by paying taxes and paying for treatments; therefore, they would not have a duty to participate based on reciprocity [32],[33]. A complete overview of the extensive discussion on whether there is a moral duty to participate in biomedical research and if this duty would be strong enough is beyond the scope of this article. However, residual tissue research is considered to generate important biomedical knowledge and the association of the research with low risks and burdens would provide an additional argument for a moral duty [31].

### Low Risks and Burdens

Research on tissue does usually not entail the same risks as research on persons. Moreover, residual samples are taken in the course of clinical care; therefore, there are no additional physical burdens involved. Consequently, many have estimated the risks of biobank research as low or even absent [3],[5]. However, the precise consequences of biobank research are sometimes difficult to predict [34] and the accompanying risks may be less clear than they appear to be [35]. Particularly, the information derived from samples can harm people [36]. There are psychological and/or social risks (e.g., genetic discrimination or stigmatization of groups, possible implications for family members, and consequences for employment or insurance possibilities) [37]–[40].

It follows that the risks involved in biobank research are tightly connected to the type of research that will be carried out on the sample, as there will be certain studies that are associated with higher risks or burdens. For example, when a researcher intends to conduct WGS, the risk of disturbing information is present. The risks are also tightly connected to the governance policy of the biobank (e.g., how the confidentiality and security measures are regulated). For example, a well-functioning Institutional Review Board (IRB), known in the European Union as a Research Ethics Committee (REC), can minimize the chance of (excessive) risks by assessing the research protocols and applying ways of risk management [3],[12].

### Compatible with Autonomy

Usually, a distinction is made between negative and positive autonomy. Negative autonomy is commonly understood as the right of a person to make personal decisions without undue influence or coercion from others [41]. Positive autonomy refers to the more “thick” interpretation of autonomy and entails the ability to take control over one's life and to live according to one's values and beliefs. In a positive account, autonomy is associated with concepts like self-expression and self-determination [41].

Within an opt-out procedure potential participants can be sufficiently informed and they can still make a personal choice whether they want to participate or not [42],[43]. Therefore, an opt-out method could be an adequate and sufficient way to respect autonomy [44]. However, this again depends on the exact implementation of the procedure. Firstly, people should be made aware of inclusion of their residual tissue as the default position. Secondly, they should receive adequate information about this and have easy access to additional information. Furthermore, they should have an accessible way to object to participation and this should be adequately registered [42],[45],[46]. For example, it is difficult to maintain that negative autonomy is respected when residual tissue is included without people knowing this. Also, when people do not receive understandable information, they will not be able to judge whether participation corresponds with the idea(l)s they have and positive autonomy will therefore not be fostered. It has been shown that, at present, there is (too) low awareness of people about the use of their residual tissue in research [47],[48] and biobanks [49].

### Positive Public Attitude

In general, people seem to have a positive attitude towards the use of their residual tissue for biomedical research, as they consider it to be an important goal [47],[50]–[53]. Some argue that because of this, an opt-out method is best suited for biobank research [44].

However, a positive public attitude towards the use of residual tissue does not necessarily signify support for opt-out procedures. In addition, people themselves have expressed that although they emphasize participation, they expressed a preference for an opt-in procedure over an opt-out procedure [54],[55].

Also, there is considerable variation in the views of the public [56]. It has been suggested that in general patients have a more positive attitude towards biobank research than the general public [57]. More specific, it has been shown that cancer patients preferred a thick opt-out procedure over an opt-in [58],[59]. In addition, surgical waste was regarded very differently compared to healthy tissue. There was less concern about how unhealthy tissue was used after removal [48]. Therefore, although a positive attitude may be shared by many, it cannot be generalized [60]. The opinion of the majority refers to democracy and should not be confused with autonomy [61],[62]. When the majority approves inclusion of leftover tissue, this should not deprive others from their right to make an autonomous decision [62].

## Arguments in Favor of an Opt-In Method for Residual Samples

### Respect for Negative Autonomy

An opt-in procedure requires an act of consent from the individual before the proposed action will be carried out. Due to this requirement, respect for negative autonomy would be safeguarded. This will be of even greater importance when the participant can be considered the owner of the removed tissue—but who (if anyone) owns human tissue, once separated from the body, is an ongoing topic of debate [51],[63],[64].

Even so, one of the main concerns with the opt-out method is the possibility of including samples from people without their knowledge and possibly against their wishes. Even when a participant is adequately protected against excessive risk, he or she can still be wronged when the decision about the inclusion of their residual tissue was taken from him or her [5],[65]. It would be misleading not to actively inform people about inclusion as the default position, as it would be reasonable for people to assume that residual tissue will be discarded. In an opt-out method it is assumed that people are aware of their inclusion, understand the information, and will take action if they do not want to participate [66]. As there is no evidence that people are willing to participate, it has been stated that opt-out, in principle, can never be considered as actual consent [67].

### Fostering Positive Autonomy

To foster positive autonomy, non-interference is insufficient; one really needs to stimulate autonomous decision making. For example, when the research may generate relevant research results, mere non-interference is insufficient for adequate decision-making. Earlier we have defended a qualified disclosure policy, where different packages of individual research results are presented [68]. Opt-in will be a more suitable procedure to discuss such a policy.

### Scientific Citizenship

Scientific citizenship is in line with the fostering of positive autonomy. It refers to a societal ideal where citizens are well informed and well-equipped to make decisions concerning scientific research. The engagement of citizens would lead to better protection and promotion of their interests. Providing participants with the opportunity to reflect on their participation and to act on that can be a way to stimulate scientific citizenship [69]. It has been noted that there are different levels of citizen participation—ranging from manipulation to actual citizen control [70]. Although an opt-in procedure seems to be more suited to stimulate citizen control (which is perceived as the highest level of participation), an opt-in method does not necessarily safeguard citizen participation as such.

### Protection of Researcher

Consent is usually considered an instrument to protect the research participant and respect his or her autonomy. However, consent can also be viewed as a means to (legally) protect the researcher [60],[71]. By obtaining consent from the participant, the responsibility of the acceptance of the entailed risks and burdens shifts (partly) from the researcher towards the participant. This may limit the liability of the researcher. Since only the opt-in procedure provides proof of consent, this method is more suited to protect the researcher.

### Public Trust

In order to conduct biomedical research public trust is indispensable. Public support is needed to facilitate research projects [72]. As noted before, distrust may result in high opt-out rates. Public distrust can be the result of people who are unaware of inclusion as the default position. Since people are not included without their explicit consent, an opt-in method is most likely to promote public trust. However, since within a thick opt-out procedure people are made aware of inclusion as the default position, public trust can be warranted as well.

## Concluding Remarks

Taking stock of the arguments in favor and against opt-in and opt-out procedures for the inclusion of residual tissue in biobanks (see Table 1), we conclude that an opt-out procedure needs to fulfill certain conditions in order to be an appropriate method to include leftover material. Public trust and respect for negative autonomy can only be sufficiently protected and warranted within an opt-out procedure when (1) awareness is raised among people about inclusion of residual tissue as the default position, (2) adequate information is provided, and (3) a genuine possibility to object is presented and objections are adequately registered. In addition, although not a characteristic of the opt-out method, another condition that needs to be fulfilled is adequate governance of the biobank in order to protect participants (e.g., to ensure confidentiality). An IRB or equivalent committee should monitor the distribution of tissue by assessing research protocols. Adequate measures should be taken to fulfill these requirements. After all, an opt-out procedure should not result in the exploitation of people's ignorance. Patients need to be actively, preferably personally, informed about the opt-out procedure [59]. Merely putting posters or leaflets in a waiting room will be insufficient. In this information there should be a description of the governance of the biobank (e.g., general information about the type of research, the identifiability of the tissue, etc.). Empirical research is needed to evaluate the implemented opt-out method.

**Table 1:** Arguments in favor opt-in and opt-out procedures.

Arguments in Favor of Opt-Out	Arguments in Favor of Opt-In
Scientific advantage – Opt-out procedures are associated with high participation rates	Respect for negative autonomy – Within an opt-in procedure, an act of consent from the individual before the proposed action will be carried out is required
Lower costs – Opt-out procedures are associated with lower costs	Fostering positive autonomy – An opt-in procedure is more suitable to stimulate autonomous decision making
Moral duty to participate – A moral duty to participate could provide an argument for a less strict consent procedure	Scientific citizenship – With an opt-in procedure, active and informed citizen participation is promoted since action is required, hence scientific citizenship is stimulated
Low risks and burdens – Biobank research with residual tissue is associated with no additional physical burdens or risks	Protection of researcher – Only the opt-in procedure provides proof of consent; therefore, this method is more suited to protect the researcher
Compatible with autonomy – Within a thick opt-out procedure potential participants can be sufficiently informed and they can still make a personal choice whether they want to participate or not	Public trust – Since people are not included without their explicit consent, an opt-in method may be more likely to promote public trust
Positive public attitude – Studies indicate a positive public attitude towards the use of residual tissue for scientific biomedical research	

An opt-out procedure that does not satisfy these conditions can be called a “thin” opt-out procedure and is clearly insufficient. An opt-out method that does fulfill the conditions can be classified as a “thick” opt-out procedure. In practice, the dichotomy between a thick opt-out and an opt-in procedure may be less stark than usually suggested. To our best knowledge, no studies have examined the exact financial differences between a thick opt-out and an opt-in procedure, but we expect this difference to be relatively small. However, it is also reasonable to assume that an opt-out will involve less administrative burden. Moreover, even if they may not differ that much in practice, they do differ in the underlying moral message. Opt-out as the default position sends the moral message that allowing your residual tissue to be used for research is the right thing to do. In contrast, with an opt-in procedure this appears more like an extraordinary act.

Secondly, we conclude that the question is not whether in general an opt-in or opt-out procedure is most suitable for residual tissue, but in which specific cases one or the other is appropriate. Residual tissue is a collective term for a diversity of samples. In addition, a wide variety of research types can be conducted on them. Hence, the appropriate method to include the tissue is context-specific and it would be too simplistic to treat all situations alike. We suggest at least four situations that would require an opt-in procedure. Firstly, there are certain types of research that are associated with increased burdens or higher risks (e.g., increased psychological or social risks associated with genetic information from WGS). When the risks or burdens of the proposed research increase, an opt-out method is no longer sufficient. Secondly, in some studies controversial and/or high-impact techniques are involved (e.g., when an immortal cell-line is derived or when chimaeras are created) [73]. As these types of research are sensitive and difficult to explain adequately in the general research information, an opt-out procedure would be insufficient. Thirdly, when sensitive cells or tissues are used (e.g., gametes), a more extensive consent procedure would be appropriate [74]. Lastly, for certain groups of vulnerable patients an opt-in method is required (e.g., psychiatric patients). For this group, the competency to understand the presented information needs to be evaluated before tissue can be included [75]. It should be the role of an independent IRB to determine when an opt-in procedure is required. Although a comprehensive overview of the situations that require an opt-in procedure has not been given, we submit that the type of tissue and research affect the appropriate consent procedure. Further discussion is needed to formulate the amount of risks and burdens, the nature of the techniques, the types of tissue, and the groups of vulnerable patients that would require an opt-in procedure. As the appropriate method is context-specific, the role of an opt-out procedure can differ between institutions and can change over time when developments in research alter the biomedical field. For instance, when a biobank intends to facilitate WGS for all samples, an opt-out method will be unfeasible. However, although the use of WGS is increasing, at the moment straightforward research still plays an important role in the scientific landscape and it seems reasonable to assume that this will continue to exist in the near future.

A third comment can be made about a consequence of the inclusion of residual tissue with a thick opt-out method. The drawback of this proposal is that researchers will have to re-contact participants when they want to conduct certain types of research (e.g., WGS, immortal cell lines) to ask additional consent. As there can be years between the inclusion of a residual sample and the actual research that will be conducted on them, it may be difficult to approach people. However, the distress that is caused to people by re-contacting them will probably decrease in a thick opt-out procedure as they will be aware of the inclusion of their residual tissue and the possibility that they will be contacted for specific research. In addition, introducing an opt-in procedure for all residual tissue research will not solve the dilemma of re-consent. Even within a broad opt-in consent procedure, it is impossible to discuss all the research possibilities that can be conducted on a sample. Previously described situations (i.e., high risks and controversial techniques) will therefore require a specific opt-in procedure as well. Hence, re-consent will also be necessary when an opt-in consent procedure is adopted for the inclusion of all residual tissue [8]. Moreover, introducing an opt-in procedure for all residual tissue research is at least at this moment overly restrictive and likely to hamper basic biomedical research unnecessarily.

In summary, an opt-out procedure is only appropriate when certain conditions are fulfilled; hence, we propose a thick opt-out method. The appropriateness of a thick opt-out method or an opt-in method is context specific. Further interdisciplinary debate is needed to determine when to opt-in or opt-out.

**Glossary**

**Biobank**: a collection of human biological samples stored for medical-scientific research purposes, usually linked to phenotypic data

**Opt-in procedure**: a procedure where a person explicitly expresses his or her consent

**Opt-out procedure**: a procedure where inaction is treated as a signal of consent

**Residual tissue**: tissue that was taken in the course of clinical care and is leftover

**Thick opt-out procedure**: an opt-out procedure *with* the fulfillment of the following conditions: (1) awareness is raised about the opt-out procedure (2) adequate information is provided (3) a genuine possibility to object is presented and objections are adequately registered

**Thin opt-out procedure**: an opt-out procedure *without* the fulfillment of the three conditions

## Abbreviations

IRB: Institutional Review Board
REC: research ethics committee
WGS: whole genome sequencing

## References

[1] Organisation for Economic Co-Operation and Development (2009) OECD guidelines on human biobanks and genetic research databases. Paris: OECD.

[2] Council of Europe, Committee of Ministers (2006) Recommendation Rec(2006)4 of the Committee of ministers to member states on research on biological materials of human origin.

[3] Coebergh JW, van Veen EB, Vandenbroucke JP, van Diest P, Oosterhuis W (2006) One-time general consent for research on biological samples: opt out system for patients is optimal and endorsed in many countries. BMJ 332: 665. 10.1136/bmj.332.7542.665 PMC1403227 16543343

[4] Furness PN (2006) One-time general consent for research on biological samples: good idea, but will it happen? BMJ 332: 665. 10.1136/bmj.332.7542.665-a PMC1403254 16543342

[5] Eriksson S, Helgesson G (2005) Potential harms, anonymization, and the right to withdraw consent to biobank research. Eur J Hum Genet 13: 1071–1076. 15986039 10.1038/sj.ejhg.5201458

[6] Heeney C, Hawkins N, de Vries J, Boddington P, Kaye J (2011) Assessing the privacy risks of data sharing in genomics. Public Health Genomics 14: 17–25. 20339285 10.1159/000294150 PMC2872768

[7] Sandor J, Bard P, Tamburrini C, Tannsjo T (2011) The case of biobank with the law: between a legal and scientific fiction. J Med Ethics 38: 347–350. 21947810 10.1136/jme.2010.041632

[8] Greely HT (2007) The uneasy ethical and legal underpinnings of large-scale genomic biobanks. Annu Rev Genomics Hum Genet 8: 343–364. 17550341 10.1146/annurev.genom.7.080505.115721

[9] Beyleveld D, Buchanan JA (2007) Consent in the law. Oxford and Portland, Oregon: Hart publishing. 187–227.

[10] Council for International Organizations of medical Sciences (CIOMS) in collaboration with the World Health Organisation (WHO) (2008) International ethical guidelines for epidemiological studies.

[11] van Veen EB, Riegman PH, Dinjens WN, Lam KH, Oomen MH, et al (2006) TuBaFrost 3: regulatory and ethical issues on the exchange of residual tissue for research across Europe. Eur J Cancer 42: 2914–2923. 17029786 10.1016/j.ejca.2006.04.028

[12] Stjernschantz FJ, Hansson MG, Eriksson S (2011) Biobank research: who benefits from individual consent? BMJ 343: d5647. 21972296 10.1136/bmj.d5647

[13] Stjernschantz FJ, Hansson MG, Eriksson S (2011) Authors' reply to Sheehan. BMJ 343: d6901.

[14] Elger B, Biller-Andorno N, Mauron A, Capron A (2008) Ethical issues in governing biobanks: global perspectives. London: Ashgate. 39–56 and pp. 89–120.

[15] Gefenas E, Dranseika V, Serepkaite J, Cekanauskaite A, Caenazzo L, et al (2012) Turning residual human biological materials into research collections: playing with consent. J Med Ethics 38: 351–355. 22408238 10.1136/medethics-2011-100113

[16] Johnsson L, Hansson MG, Eriksson S, Helgesson G (2008) Patients' refusal to consent to storage and use of samples in Swedish biobanks: cross sectional study. BMJ 337: a345. 18617496 10.1136/bmj.a345 PMC2656925

[17] Pulley J, Clayton E, Bernard GR, Roden DM, Masys DR (2010) Principles of human subjects protections applied in an opt-out, de-identified biobank. Clin Transl Sci 3: 42–48. 20443953 10.1111/j.1752-8062.2010.00175.x PMC3075971

[18] Ellis SD, Bertoni AG, Bonds DE, Clinch CR, Balasubramanyam A, et al (2007) Value of recruitment strategies used in a primary care practice-based trial. Contemp Clin Trials 28: 258–267. 17030154 10.1016/j.cct.2006.08.009 PMC3760001

[19] Green LA, Lowery JC, Kowalski CP, Wyszewianski L (2006) Impact of institutional review board practice variation on observational health services research. Health Serv Res 41: 214–230. 16430608 10.1111/j.1475-6773.2005.00458.x PMC1681539

[20] Junghans C, Feder G, Hemingway H, Timmis A, Jones M (2005) Recruiting patients to medical research: double blind randomised trial of “opt-in” versus “opt-out” strategies. BMJ 331: 940. 16157604 10.1136/bmj.38583.625613.AE PMC1261191

[21] Metcalfe C, Martin RM, Noble S, Lane JA, Hamdy FC, et al (2008) Low risk research using routinely collected identifiable health information without informed consent: encounters with the Patient Information Advisory Group. J Med Ethics 34: 37–40. 18156520 10.1136/jme.2006.019661 PMC2762744

[22] Laurie G (2008) Consent for Biobanking. Lack of dissent when opting in doesn't necessarily support “opt out”. BMJ 337: a337. 18617495 10.1136/bmj.a337

[23] HUGO Ethics Committee (1998) Statement on DNA sampling: control and access. London: HUGO Ethics Committee. 12817580

[24] UNESCO (2011) Casebook on benefit and harm, Bioethics Core Curriculum Casebook Series, No. 2. Paris: UNESCO. 140.

[25] Harris J (2005) Scientific research is a moral duty. J Med Ethics 31: 242–248. 15800367 10.1136/jme.2005.011973 PMC1734128

[26] Chan S, Harris J (2009) Free riders and pious sons–why science research remains obligatory. Bioethics 23: 161–171. 18445091 10.1111/j.1467-8519.2008.00648.x PMC3579232

[27] van Diest PJ (2002) No consent should be needed for using leftover body material for scientific purposes. BMJ 325: 648–651. 12242180

[28] Chadwick R, Berg K (2001) Solidarity and equity: new ethical frameworks for genetic databases. Nat Rev Genet 2: 318–321. 11283704 10.1038/35066094

[29] Human Genetics Commission (2002) Inside information: balancing interests in the use of personal genetic data. 35–46. 12379989

[30] Prainsack B, Buyx A (2011) Solidarity: reflections on an emerging concept in bioethics. London: Nuffield Council on Bioethics.

[31] Schaefer GO, Emanuel EJ, Wertheimer A (2009) The obligation to participate in biomedical research. JAMA 302: 67–72. 19567441 10.1001/jama.2009.931 PMC2763192

[32] Brassington I (2007) John Harris' argument for a duty to research. Bioethics 21: 160–168. 17845487 10.1111/j.1467-8519.2007.00539.x

[33] Shapshay S, Pimple KD (2007) Participation in biomedical research is an imperfect moral duty: a response to John Harris. J Med Ethics 33: 414–417. 17601870 10.1136/jme.2006.017384 PMC2598131

[34] Solbakk JH, Holm S, Hofmann B (2009) The ethics of research biobanking. LLC: Springer Science+Business Media 3–24.

[35] Maschke KJ (2006) Alternative consent approaches for biobank research. Lancet Oncol 7: 193–194. 16510329 10.1016/S1470-2045(06)70590-3

[36] Savulescu J (2002) No consent should be needed for using leftover body material for scientific purposes. BMJ 325: 648–651. 12269316

[37] Medical Research Council (MRC) (2001) Human tissue and biological samples for use in research: operational and ethical guidelines.

[38] Bredenoord AL, Kroes HY, Cuppen E, Parker M, van Delden JJ (2011) Disclosure of individual genetic data to research participants: the debate reconsidered. Trends Genet 27: 41–47. 21190750 10.1016/j.tig.2010.11.004

[39] Caulfield T, McGuire AL, Cho M, Buchanan JA, Burgess MM, et al (2008) Research ethics recommendations for whole-genome research: consensus statement. PLoS Biol 6: e73 doi:10.1371/journal.pbio.0060073. 18366258 10.1371/journal.pbio.0060073 PMC2270329

[40] Council for International Organizations of Medical Sciences (CIOMS) (2002) International ethical guidelines for biomedical research involving human subjects. 14983848

[41] Berlin I (1969) Four essays on liberty. Oxford: Oxford University Press. 166–217.

[42] Clark AM, Findlay IN (2005) Attaining adequate consent for the use of electronic patient records: an opt-out strategy to reconcile individuals' rights and public benefit. Public Health 119: 1003–1010. 16185734 10.1016/j.puhe.2005.08.013

[43] Hewison J, Haines A (2006) Overcoming barriers to recruitment in health research. BMJ 333: 300–302. 16888308 10.1136/bmj.333.7562.300 PMC1526952

[44] Johnsson L, Hansson MG, Eriksson S, Helgesson G (2008) Opt-out from biobanks better respects patients' autonomy. BMJ 337: a1580. 18784161 10.1136/bmj.a1580

[45] Adams T, Budden M, Hoare C, Sanderson H (2004) Lessons from the central Hampshire electronic health record pilot project: issues of data protection and consent. BMJ 328: 871–874. 15073070 10.1136/bmj.328.7444.871 PMC387480

[46] FEDERA (2011) Gedragscode 2011: Verantwoord omgaan met lichaamsmateriaal ten behoeve van wetenschappelijk onderzoek [in Dutch].

[47] Pulley JM, Brace MM, Bernard GR, Masys DR (2008) Attitudes and perceptions of patients towards methods of establishing a DNA biobank. Cell Tissue Bank 9: 55–65. 17960495 10.1007/s10561-007-9051-2

[48] Medical Research Council (MRC) (2001) Public perceptions of the collection of human biological samples.

[49] Gaskell G, Gottweis H (2011) Biobanks need publicity. Nature 471: 159–160. 21390108 10.1038/471159a

[50] Brothers KB, Morrison DR, Clayton EW (2011) Two large-scale surveys on community attitudes toward an opt-out biobank. Am J Med Genet Part A 155: 2982–2990. 10.1002/ajmg.a.34304 PMC3222722 22065592

[51] Bryant RJ, Harrison RF, Start RD, Chetwood AS, Chesshire AM, et al (2008) Ownership and uses of human tissue: what are the opinions of surgical in-patients? J Clin Pathol 61: 322–326. 18256118 10.1136/jcp.2007.053173

[52] Wendler D (2006) One-time general consent for research on biological samples. BMJ 332: 544–547. 16513715 10.1136/bmj.332.7540.544 PMC1388140

[53] Cousins G, McGee H, Ring L, Conroy R, Kay E, et al (2005) Public perceptions of biomedical research: a survey of the general population in Ireland.

[54] Simon CM, L'heureux J, Murray JC, Winokur P, Weiner G, et al (2011) Active choice but not too active: public perspectives on biobank consent models. Genet Med 13: 821–831. 21555942 10.1097/GIM.0b013e31821d2f88 PMC3658114

[55] Ludman EJ, Fullerton SM, Spangler L, Brown Trinidad S, Fujii MM, et al (2010) Glad you asked: participants' opinions of re-consent for dbGaP data submission. J Empir Res Hum Res Ethics 5: 9–16. 20831417 10.1525/jer.2010.5.3.9 PMC3071850

[56] Gottweis H, Chen H, Starkbaum J (2011) Biobanks and the phantom public. Hum Genet 130: 433–440. 21773770 10.1007/s00439-011-1065-y

[57] Johnsson L, Helgesson G, Rafnar T, Halldorsdottir I, Chia KS, et al (2010) Hypothetical and factual willingness to participate in biobank research. Eur J Hum Genet 18: 1261–1264. 20648060 10.1038/ejhg.2010.106 PMC2987483

[58] Vermeulen E, Schmidt MK, Aaronson NK, Kuenen M, van der Valk P, et al (2009) Opt-out plus, the patients' choice: preferences of cancer patients concerning information and consent regimen for future research with biological samples archived in the context of treatment. J Clin Pathol 62: 275–278. 19017681 10.1136/jcp.2008.061069

[59] Vermeulen E, Schmidt MK, Aaronson NK, Kuenen M, Baas-Vrancken Peeters MJ, et al (2009) A trial of consent procedures for future research with clinically derived biological samples. Br J Cancer 101: 1505–1512. 19861997 10.1038/sj.bjc.6605339 PMC2778511

[60] Hoeyer K (2010) Donors perceptions of consent to and feedback from biobank research: time to acknowledge diversity? Public Health Genomics 13: 345–352. 19940458 10.1159/000262329

[61] Hunter D (2006) One-time general consent for research on biological samples: autonomy and majority rules have been misunderstood. BMJ 332: 665–666. 10.1136/bmj.332.7542.665-b PMC1403216 16543341

[62] Caulfield T (2007) Biobanks and blanket consent: the proper place of the public good and public perception rationales. Kings Law J 18: 209–226.

[63] Haga SB, Beskow LM (2008) Ethical, legal, and social implications of biobanks for genetics research. Adv Genet 60: 505–544. 18358331 10.1016/S0065-2660(07)00418-X

[64] Glantz L, Roche P, Annas GJ (2008) Rules for donations to tissue banks–what next? N Engl J Med 358: 298–303. 18199870 10.1056/NEJMhle074597

[65] Clayton EW, Steinberg KK, Khoury MJ, Thomson E, Andrews L, et al (1995) Informed consent for genetic research on stored tissue samples. JAMA 274: 1786–1792. 7500511

[66] Petrini C (2010) “Broad” consent, exceptions to consent and the question of using biological samples for research purposes different from the initial collection purpose. Soc Sci Med 70: 217–220. 19853341 10.1016/j.socscimed.2009.10.004

[67] Shickle D (2006) The consent problem within DNA biobanks. Stud Hist Philos Biol Biomed Sci 37: 503–519. 16980191 10.1016/j.shpsc.2006.06.007

[68] Bredenoord AL, Onland-Moret NC, van Delden JJM (2011) Feedback of individual genetic results to research participants: in favor of a qualified disclosure policy. Hum Mutat 32: 861–867. 21538687 10.1002/humu.21518

[69] Árnason V (2009) Scientific citizenship, benefit, and protection in population-based research. In: Solbakk JH, Holm S, Hofmann B, editors. The ethics of research biobanking. LLC: Springer Science+Business Media. 131–141.

[70] Arnstein SR (1969) A ladder of citizen particiaption. Journal of the American Insitute for Planners 35: 216–224.

[71] Levine RJ (2003) Consent issues in human research. In: Emanuel EJ, Crouch RA, Arras JD, Moreno JD, Grady C, editors. Ethical and regulatory aspects of clinical research: readings and commentary. John Hopkins University Press. 197–201.

[72] Tutton R, Kaye J, Hoeyer K (2004) Governing UK Biobank: the importance of ensuring public trust. Trends Biotechnol 22: 284–285. 15158057 10.1016/j.tibtech.2004.04.007

[73] Aalto-Setälä K, Conklin BR, Lo B (2009) Obtaining consent for future research with induced pluripotent cells: opportunities and challenges. PLoS Biol 7: e42 doi:10.1371/journal.pbio.1000042. 19243222 10.1371/journal.pbio.1000042 PMC2652391

[74] Parker L (2011) Using human tissue: when do we need consent? J Med Ethics 37: 759–761. 21873308 10.1136/medethics-2011-100043

[75] van der Baan FH, Bernabe RD, Bredenoord AL, Gregoor JG, Meynen G, et al (2011) Consent in psychiatric biobanks for pharmacogenetic research. Int J Neuropsychopharmacol 21: 1–6. 10.1017/S146114571200048X 22607776

